# To the American Medical Association

**Published:** 1867-05

**Authors:** A. Stillé, Gustav C. E. Weber

**Affiliations:** President; Secretary


### To the American Medical Association.

The Convention of Teachers of the Medical Colleges, convened by your Committee in the City of Cincinnati, May 3d, 1867, adopted the following resolution:

*Resolved*, "That a copy of the Resolutions adopted by this Convention, certified to by its officers, be transmitted to the American Medical Association, at its next Session."

In obedience, therefore, to this action, we, the President and Secretary of said Convention, beg leave herewith to transmit the following:

"*Resolved*—1st. That every student applying for matriculation in a Medical College, shall be required to show, either by satisfactory certificate, or by a direct examination by a Committee of the Faculty, that he possesses a thorough knowledge of the common English branches of Education, including the first series of Mathematics, elements of Natural Sciences, and a sufficient knowledge of Latin and Greek to understand the technical terms of the profession; and that the certificate presented on the result of the examination thus required, be regularly filed as a part of the records of each Medical College.

2d. That every medical student be required to study four full years, including three regular annual courses of Medical College instruction, before being admitted to an examination for the degree of Doctor of Medicine.

3d. That the minimum duration of a regular annual lecture term, or course of Medical College instruction, shall be six calendar months.

4th. That every Medical College shall embrace in its curriculum the following branches, to be taught by not less than nine Professors, namely: Descriptive Anatomy, including Dissections; Inorganic Chemistry, Materia Medica, Organic Chemistry and Toxicology; General Pathology, Therapeutics, Pathological Anatomy and Public Hygiene; Surgical Anatomy and Operation of Surgery; Medical Jurisprudence and Medical Ethics; Practice of Medicine, Practice of Surgery, Obstetrics, and Diseases of Women and Children; Clinical Medicine and Clinical Surgery. And that these several branches shall be divided into three groups or series, corresponding with the three courses of Medical College instruction required.

The first, or FRESHMAN SERIES, shall embrace Descriptive Anatomy and Practical Dissections; Physiology and Histology; Inorganic Chemistry, Materia Medica and Therapeutics. To these the attention of the student shall be mainly restricted during his first course of Medical College Instruction, and in these he shall submit to a thorough examination, by the proper members of the Faculty, at its close, and receive a certificate indicating the degree of his progress.

The second, or JUNIOR SERIES, shall embrace Organic Chemistry and Toxicology; General Pathology, Morbid Anatomy and Public Hygiene; Surgical Anatomy Operations of Surgery; Medical Jurisprudence and Medical Ethics. To these the attention of the medical student shall be directed during his second course of Medical College Instruction, and in them he shall be examined at the close of his second course, in the same manner as after the first.

The third, or SENIOR SERIES, shall embrace Practical Medicine; Practical Surgery, Obstetrics, and Diseases peculiar to women and children, with Clinical Medicine and Clinical Surgery in Hospital. These shall occupy the attention of the student during his third course of College instruction, and at its close he shall be eligible to a general examination for the Degree of Doctor of Medicine.

The instruction in the three series is to be given simultaneously, and to continue throughout the whole of each annual College term; each student attending the lectures on such branches as belong to his period of progress in study, in the same manner as the Sophomore, Junior and Senior Classes, each pursue their respective studies simultaneously throughout the Collegiate Year, in all our Literary Colleges.

5th. That every Medical College should immediately adopt some effectual method of ascertaining the actual attendance of students, upon its lectures and other exercises, and at the close of each session, of the attendance of the student a certificate, specifying the time and the courses of instruction actually attended, should be given, and such certificate only should be received by other Colleges as evidence of such attendence.

6th. That a Committee of Five be appointed by the President, whose duty it shall be to present the several propositions adopted by the Convention, to the Trustees and Faculties of all the Medical Colleges in this country, and solicit their definite action thereon, with a view to the early and simultaneous practical adoption of the same throughout the whole country. And that the Committee be authorized to call another Convention whenever deemed advisable."

Respectfully Forwarded,

A. STILLÉ, M. D., PRESIDENT.

GUSTAV C. E. WEBER, M. D., SECRETARY.

